# Supplementary material for: Comparison of Two Different Sedation Protocols during Transvaginal Oocyte Retrieval: Effects on Propofol Consumption and IVF Outcome: A Prospective Cohort Study
Source: J Clin Med. 2021 Mar 1;10(5):963. doi: 10.3390/jcm10050963 (PMC7957650; doi:10.3390/jcm10050963)
Supplement: Supplementary file 1 [file jcm-10-00963-s001.pdf]

**Figure S1. Sample size calculation**

For the calculation of sample size, G*Power 3.0.10 software was used. The mathematical formula is the following:

$$T = -\ln\left[\frac{\binom{n_1}{x_1}\binom{n_2}{x_2}}{\binom{N}{m}}\right]$$

The following output was produced:

| | | | |
|---|---|---|---|
| **Analysis:** | A priori: Compute required sample size | | |
| **Input:** | Tail(s) | = | Two |
| | Effect size d | = | 0.5 |
| | α err prob | = | 0.05 |
| | Power (1−β err prob) | = | 0.90 |
| | Allocation ratio N2/N1 | = | 1 |
| **Output:** | Noncentrality parameter δ | = | 3.278719 |
| | Critical t | = | 1.974017 |
| | Df | = | 170 |
| | Sample size group 1 | = | 86 |
| | Sample size group 2 | = | 86 |
| | Total sample size | = | 172 |
| | Actual power | = | 0.903230 |

| | | | |
|---|---|---|---|
| **Analysis:** | Post hoc: Compute achieved power | | |
| **Input:** | Tail(s) | = | Two |
| | Effect size d | = | 1.2473714 |
| | α err prob | = | 0.05 |
| | Sample size group 1 | = | 36 |
| | Sample size group 2 | = | 36 |
| **Output:** | Noncentrality parameter δ | = | 5.292149 |
| | Critical t | = | 1.994437 |
| | Df | = | 70 |
| | Power (1−β err prob) | = | 0.999440 |
